# Host genotype and environment shape rhizosphere and root microbiome composition of pecan rootstocks

**DOI:** 10.3389/frmbi.2026.1778537

**Published:** 2026-05-15

**Authors:** Paul Oladimeji Gabriel, Ciro Velasco-Cruz, Jennifer J. Randall

**Affiliations:** 1 Entomology, Plant Pathology, and Weed Science, New Mexico State University, Las Cruces, NM, United States; 2 Molecular Biology and Interdisciplinary Life Sciences, New Mexico State University, Las Cruces, NM, United States; 3 Agriculture Economics and Agriculture Business, New Mexico State University, Las Cruces, NM, United States

**Keywords:** microbial diversity, microbiome, pecan, rhizosphere, rootstock

## Abstract

The rhizosphere and root-associated microbiomes play a crucial role in nutrient acquisition, stress tolerance, and overall plant performance. However, little is known about how microbial communities assemble and shift across environments in pecan (*Carya illinoinensis*). In this study, we compared the bacterial and fungal community compositions in the roots and rhizosphere of four pecan clonal rootstocks (NMU03, NMU04, NMU05, and NMU155) cultivated under greenhouse conditions, as well as their subsets that were subsequently transplanted to the field. Amplicon sequencing of 16S rRNA and ITS regions revealed significant differences in microbial diversity and taxonomic composition across environments and genotypes. Bacterial assemblages in greenhouse roots were typically dominated by a few families (e.g., *Burkholderiaceae, Rhodanobacteraceae*, and unclassified taxa). In contrast, field samples exhibited broader taxonomic distributions, with families such as *Xanthobacteraceae, Haliangiaceae*, and *Geminicoccaceae* emerging as dominant members. Fungal OTU abundance was consistently higher than bacterial abundance across all genotypes, likely reflecting mutualistic associations with mycorrhizal fungi, such as those in the *Elaphomycetaceae family*. Interestingly, *Aspergillaceae* dominated greenhouse and field fungal communities, suggesting ecological adaptability and potential contributions to plant stress tolerance. Comparisons with earlier greenhouse studies revealed that while some signature core microbiome members were retained following transplantation from the greenhouse to the field, the abundance of others decreased, highlighting successional shifts in community structure driven by environmental transitions. Together, these findings demonstrate the dynamic, genotype and environment-specific structuring of pecan microbiomes and highlight the importance of microbiome-informed breeding strategies to improve plant-microbe associations under variable growth conditions among pecan breeders.

## Introduction

Soil is a natural biological resource that harbors diverse ecosystems, many organisms, and essential nutrients. Among these, soil-bound microorganisms represent one of the most abundant and diverse groups of organisms, and their presence and balanced interaction within the soil ecosystem play a critical role in maintaining plant health and ecosystem function (Joos and De Tender, 2022). These microbial communities are shaped by biotic and abiotic factors, including plant hosts, climate, soil type, and management practices (Koberl et al., 2020). Plants are colonized by complex and diverse microbial communities, collectively known as the plant microbiome, which inhabit various ecological niches, including the rhizosphere (the soil surrounding the roots), the phylloplane (the leaf surfaces), and the endosphere (the internal tissues of the plant). The complex community of microorganisms inhabiting the soil-root interface plays a crucial role in shaping the plant, including nutrient acquisition and resilience to both biotic and abiotic stresses. Hence, plant microbiomes function as a dynamic extension of the plant’s genome (Solomon et al., 2024; Tharanath et al., 2024). However, the relationship between plant roots and their associated microbiomes encompasses a range of interactions, including mutualistic, phytopathogenic, commensal, and neutral associations. Therefore, while plants possess innate mechanisms for nutrient acquisition and stress response, symbiotic interactions with microorganisms often play a supportive and beneficial role under both biotic and abiotic stress conditions (Glick and Gamalero, 2021; Srivastava et al., 2021).

Controlled greenhouse studies are crucial for understanding how the plant-associated microbiome affects host health and productivity. The ability of plants to thrive under complex microbial communities and fluctuating environmental conditions is critical for their long-term survival and productivity (Schlaeppi and Bulgarelli, 2015; Kushwaha et al., 2024). However, transplanting plant cultivars or rootstocks from their original habitat to a new environment may result in shifts in microbiome composition due to differences in local climatic and edaphic conditions. Previous research has demonstrated that the composition of the plant microbiome is shaped by multiple factors, including host genotype, developmental stage, environmental conditions, tissue type, and management practices (Redford et al., 2010; Hacquard et al., 2015; Randall et al., 2021). Understanding these interactions is vital for optimizing plant resilience and productivity in variable agricultural environments.

Pecan *(Carya illinoinensis (Wangenh.) K. Koch)* is a perennial, deciduous tree species belonging to the family *Juglandaceae* and is a specialty crop cultivated for its highly nutritious nuts. Pecan is indigenous to a broad range from Illinois, USA, to Oaxaca, Mexico. Its natural habitat includes riverbanks, floodplains, and well-drained bottomland soils, where it thrives under temperate to subtropical climates and various soil pH levels (Thompson and Grauke, 1991). As a perennial crop adapted to semi-arid and humid environments, the productivity of pecan is significantly influenced by a range of biotic and abiotic stressors, including pests and various pathogens which are common to its endemic regions (Cervantes et al., 2022; Lovell et al., 2021), hence, the pecan tree is uniquely positioned at the intersection of ecological resilience and agricultural importance.

Pecans are commonly propagated by grafting scions onto selected rootstocks to retain desirable traits and improve tree growth and development. The selection of rootstock genotypes has been shown to affect multiple agronomic traits, including plant height, trunk diameter, flowering phenology, nut yield, disease resistance, and overall tree performance (Doornbos et al., 2012; Turner et al., 2013; Wood, 2016; Wang et al., 2025).

Recent studies have demonstrated that both maternal cultivars and rootstock genotypes can significantly influence the composition and diversity of microbial communities in pecan seedlings and in the rhizosphere of mature trees (Cervantes et al., 2023; Ren et al., 2024; Wang et al., 2025). However, the dynamics and stability of these microbiomes following transplantation from controlled greenhouse conditions to field environments remain poorly understood. Understanding how microbial communities respond to such transitions is essential for advancing our understanding of plant-soil-microbe interactions and optimizing orchard establishment practices under varying environmental conditions.

This study examined the microbial communities associated with the rhizosphere soil and roots of four pecan rootstock genotypes cultivated under greenhouse conditions, as well as their corresponding subsets following transplantation into the field. Each genotype was derived from one of the following maternal cultivars: ‘*Wichita*’ and ‘*Riverside*’. The ‘*Wichita*’ cultivar is one of the most commercially important pecan varieties in semiarid production regions, due to its high nut yield, early fruiting, and consistent performance under irrigation systems (Heerema et al., 2017). In contrast, *‘Riverside’* is known for its vigorous growth, deep root development, and tolerance to salinity and alkaline soils, traits that enhance adaptability and long-term survival in semi-arid orchard environments (Cao et al., 2019). Specifically, we aim to investigate the diversity of bacterial and fungal communities associated with roots and rhizosphere soils of pecan clonal rootstocks in both greenhouse conditions and their corresponding subsets transplanted to the field. By integrating an amplicon-based sequencing approach, our research provides insight into the interactions between plants, microbes, and their environment. This approach not only deepens our understanding of microbiome adaptation across environmental gradients but also offers practical insights for microbiome-informed orchard management in semi-arid regions.

## Methods

### Plant materials and samples collection

Pecan clonal rootstock genotypes NMU03, NMU04, NMU05, and NMU155 were propagated from open pollinated seed that were germinated and subsequently propagated in tissue culture and later transplanted in potting mix. In 2018, the clonal rooted trees were removed from tissue culture conditions and planted in Lambert High-Professional high-porosity Growing Mix (Québec, Canada) that was treated with hydrogen peroxide 24 hours prior to planting. The clones were grown under quarantine conditions in a greenhouse at New Mexico State University, Las Cruces campus, maintained at 29–35 °C. These rootstock clonal rootstock genotypes are experimental and not available for commercial use. The NMU03, NMU04, and NMU05 clones were derived from seed from the ‘Wichita’ maternal cultivar. All three of these rootstocks had salinity tolerance when tested in laboratory conditions however, there were differences in these genetically different clonal genotypes in the timing of their development. NMU155 clones were derived from seed from a ‘Riverside’ maternal tree and also exhibited salinity tolerance. Likewise, a subset of these trees, were transplanted into a field plot in 2021 at the New Mexico State University Plant Science Research Center (latitude 32.199167°N, Longitude -106.741944°W). The subset was grown in the field for two years while the remainder of the plants were maintained in the quarantine greenhouse under controlled conditions. Rhizosphere (soil) and root samples were collected from both greenhouse-grown and field-transplanted clonal trees during the fall of 2023 (October). For each sample location (greenhouse and field), three biological replicates were obtained per genotype, resulting in a total of 24 samples (4 genotypes × 2 environments × 3 replicates). Samples were immediately transported on ice to the laboratory for downstream analysis. Voucher specimens for each genotype were prepared following herbarium standards and deposited at the New Mexico State University Herbarium (NMC) under the accession numbers NMC103073 (NMU03), NMC103076 (NMU04), NMC103074 (NMU05), and NMC103075 (NMU155).

### Genomic DNA extraction

Rhizosphere soil samples collected from each tree were transferred into Cole-Palmer ¾ x 2” polycarbonate vials, flash frozen in liquid nitrogen, and homogenized using the Cole-Palmer Genolyte HG-200 homogenizer (serial No. 14014). Flash-frozen root tissues were ground into a fine powder in liquid nitrogen using a pre-chilled, sterile mortar and pestle. Homogenized soil and root materials were transferred into labeled microcentrifuge tubes and stored at -20 °C until further processing. Genomic DNA was extracted from soil samples using the DNeasy PowerSoil Pro Kit (Qiagen, Hilden, Germany; #47014), following the manufacturer’s protocol with modifications as described by Stock et al. (2025; https://dx.doi.org/10.17504/protocols.io.eq2ly7domlx9/v1). Genomic DNA from root samples was extracted using the DNeasy Plant Mini Kit (Qiagen, Hilden, Germany; #69106), following the manufacturer’s standard protocol. The concentration and purity of the extracted DNA were assessed using a NanoDrop 2000 UV-Vis spectrophotometer (Thermo Fisher Scientific, Waltham, MA, USA). DNA samples were subsequently stored at -20 °C to preserve the DNA integrity for downstream analyses.

### Next-generation sequencing and microbiome analysis

DNA samples with concentrations ranging from 23.5 ng/μL to 100 ng/μL, and both A260/280 and A260/230 ratios of approximately 2.0, were submitted to the University of Minnesota Genomics Center (UMGC) in Minneapolis, MN, USA, for Illumina MiSeq sequencing. For bacterial community analysis, primers targeting the V4-V6 hypervariable region of the 16S rRNA gene were used. Fungal communities were analyzed using primers specific to the internal transcribed spacer 2 (ITS2) region. The UMGC performed Illumina DNA library preparation using compatible primers for bacterial community profiling, following a dual-indexed, two-step amplification protocol. Sequencing was conducted on an Illumina platform. For ITS amplicons, library preparation followed the Earth Microbiome Project (EMP) ITS protocol with minor modifications, as described by Gohl et al. (2016). Primer sequences are detailed in **Table 1**.

**Table 1:** Sequences of 16S and ITS2 primers used for sequencing DNA samples.

Marker gene	Target region	Primer direction	Sequence
16S rRNA	V4-V6	Forward	TCGTCGGCAGCGTCAGATGTGTATAAGAGACAGG TGCCAGCMGCCGCGGTAA
16S rRNA	V4-V6	Reverse	GTCTCGTGGGCTCGGAGATGTGTATAAGAGACAG CGACRRCCATGCANCACCT
ITS	ITS2	Forward	TCGTCGGCAGCGTCAGATGTGTATAAGAGACAGTC GATGAAGAACGCAGCG
ITS	ITS2	Reverse	GTCTCGTGGGCTCGGAGATGTGTATAAGAGACAGTC CTCCGCTTATTGATATGC

### Microbiome analysis

Microbiome analysis was conducted using QIAGEN CLC Microbial Genomics Module 24.0 within CLC Genomics Workbench 24.0.2, following established workflows with targeted modifications described by Cervantes et al. (2022). Paired-end Illumina sequences underwent quality trimming (NCBI/Sanger/Illumina 1.8+ standards), adapter removal, and length adjustment was set to a fixed length of 210 bp for 16S rRNA and ITS regions, with fragment merging distances set to 150–1000 bp. Samples were filtered to retain those with a minimum of 100 reads and a median sample depth of 25%, to balance sensitivity and noise reduction. An Operational Taxonomic Unit (OTU) was generated for novel taxa at the family level using a reference-based classification against the bacterial SILVA 16S v132 and fungal UNITE v7.2 databases, with a threshold of 99% similarity. Parameters used include: 80% taxonomy similarity threshold, minimum occurrences of 1, fuzzy match duplicates, and find best match. While chimeric sequences were removed through fragment-based alignment against reference databases, with additional exclusion of mitochondrial/chloroplast sequences in bacterial analyses. The MUSCLE tool within the Microbial Genomics Module was used to align the sequences based on a Multiple Sequence Alignment (MSA) of OTU sequences from bacterial and fungal communities. A phylogenetic tree was then constructed using the Maximum Likelihood method with the Jukes-Cantor nucleotide substitution model. The resulting phylogenetic tree was utilized for alpha diversity assessments. Rarefaction analysis was conducted at a maximum sampling depth of 5,000 reads for both communities. Alpha diversity was visualized using box plots generated in CLC Microbial Genomics Module version 24.0. Alpha diversity comparisons were measured using the non-parametric Kruskal-Wallis and Mann-Whitney tests to address skewed diversity distributions inherent in the microbiome datasets.

Microbial community composition was analyzed in R (version ≥ 4.2.0) using the *vegan* package. OTU counts were transformed to relative abundances prior to analysis. Bray-Curtis dissimilarities were calculated to quantify differences in community composition among samples. Non-metric multidimensional scaling (NMDS; k = 2) was performed using the metaMDS function to visualize variation in community structure across environments, genotypes, and sample types. Ordination fit was assessed using stress values, and Shepard plots were examined to evaluate the relationship between ordination distances and observed dissimilarities. Permutational multivariate analysis of variance (PERMANOVA) was conducted with the adonis2 function (999 permutations) to test the effects of Genotype, Environment, and their interaction. Raw sequence data were deposited in the NCBI Sequence Read Archive under BioProjects PRJNA1293544 (bacterial) and PRJNA1294893 (fungal).

## Results

### Taxonomic profiles of bacterial and fungal communities in rhizosphere soil and roots

**Figure 1:**
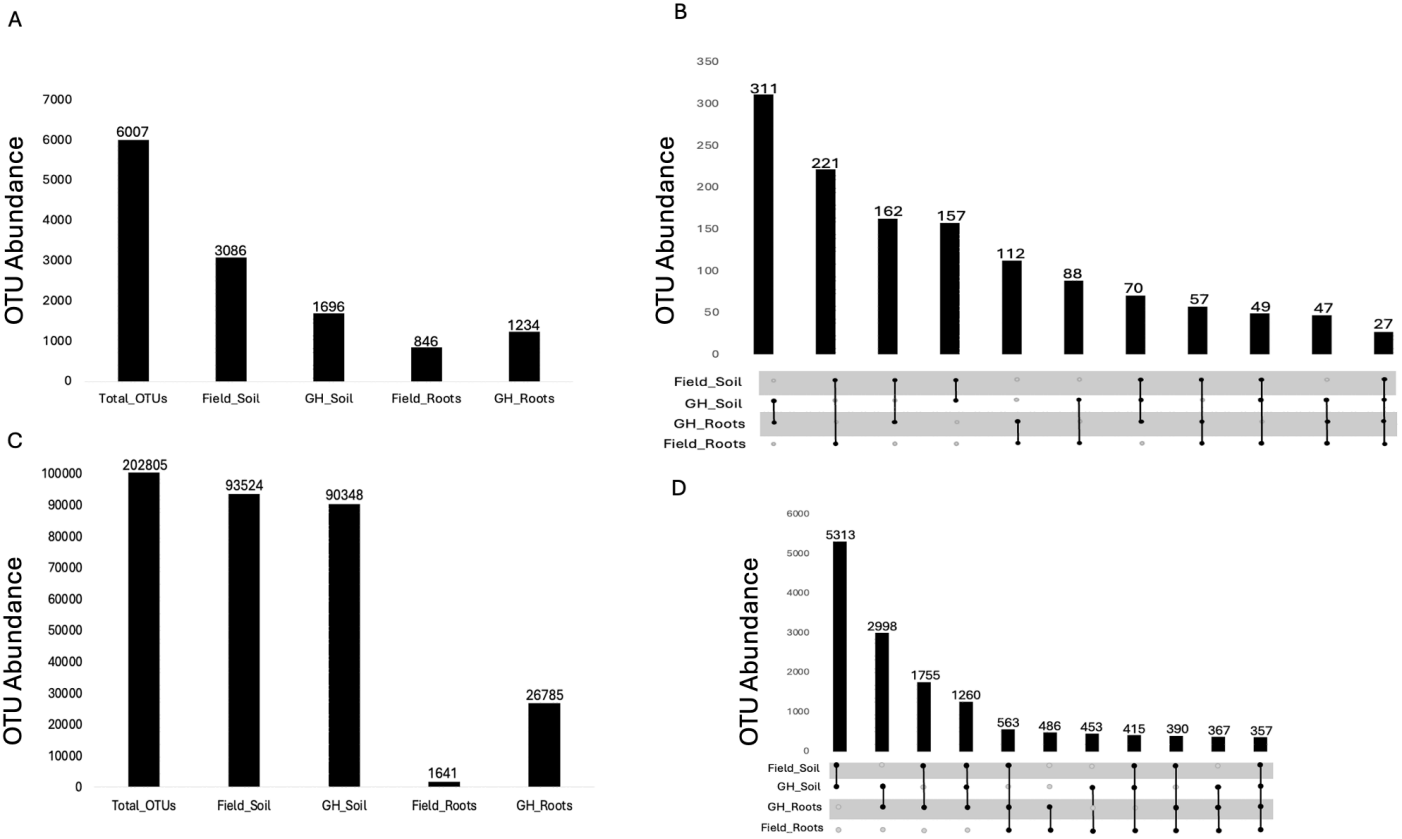
Bar chart of pecan OTU counts. Each bar chart shows the Microbiome distribution of **(A)** Bacterial population, **(B)** shared bacterial population, **(C)** fungal population, and **(D)** shared fungal population across root and soil compartments under field and greenhouse conditions. In plots B and D, each vertical bar (intersection bar) represents the number of OTUs, or taxa shared among specific sample group combinations. The connected dot matrix below the intersection bars shows which combinations of samples are being compared, with a single dot representing a unique set and connected dots representing shared membership.

Microbiome sequencing of the bacterial community yielded 6,007 OTUs after excluding sequences associated with mitochondria, chloroplasts, unknown, and ambiguous taxa. Of 6,007 identified bacterial OTUs, 3,086 were associated with field soil, whereas 1,696 were detected in greenhouse soil. The lowest number of OTUs was observed in field roots (846), compared to 1,234 OTUs identified in greenhouse roots (**Figure 1A**). Further analysis indicated that 27 OTUs were shared between all the sample types (field rhizosphere soil and roots, and greenhouse rhizosphere soil and roots), 112 OTUs were shared between field and greenhouse roots, and 157 OTUs were shared between field and greenhouse rhizosphere soils (**Figure 1B**).

MiSeq sequencing of the ITS2 region revealed substantial variation in the abundance of the fungal community across root and rhizosphere compartments of pecan clonal rootstocks under field and greenhouse conditions. Unique OTUs exhibiting non-zero abundance were quantified for each genotype. The fungal community yielded 202,805 OTUs after excluding sequences associated with unknown or ambiguous taxa. Of the 202,805 fungal OTUs, 93,524 were found in field soil, in contrast to 90,348 associated with greenhouse soil. However, similar to the bacterial OTU count, the fewest fungal OTUs were detected in field roots (1,641), whereas 26,785 were observed in greenhouse roots (**Figure 1C**).

Further analysis indicated that 357 OTUs were shared between all the sample types, 486 OTUs were shared between field and greenhouse roots, and 5,313 OTUs were shared between field and greenhouse rhizosphere soils (**Figure 1D**).

Individual pecan clonal rootstock genotypes exhibited varying bacterial OTU counts when transferred from the greenhouse to the field (**Table 2**). In the NMU03 group, 368 OTUs were detected in field-derived rhizosphere soil and root samples, whereas 216 OTUs were identified from the corresponding greenhouse samples. For the NMU04 group, 1,580 OTUs were observed in field rhizosphere soil and root samples, compared with 1,114 in greenhouse samples. In the NMU05 group, field rhizosphere soil and root samples harbored 1,631 OTUs, while 1,008 OTUs were identified in the greenhouse-derived samples. Lastly, in the NMU155 group, field rhizosphere soil and root samples contained 1,664 OTUs, whereas 1,062 OTUs were detected in the greenhouse samples.

**Table 2:** Taxonomic and aggregated family OTU counts across genotypes and environments.

Taxonomic OTU Counts				Aggregated Family OTU Counts						
Genotype	NMU03	NMU04	NMU05	NMU155	NMU03	NMU04	NMU05	NMU155
Environment	Field GH	Field GH	Field GH	Field GH	Field GH	Field GH	Field GH	Field GH
Bacteria	368 216	1580 1114	1631 1008	1664 1062	271 143	695 564	714 547	695 525
Fungi	365 344	34676 44832	39618 56481	31430 24953	170 130	385 454	469 567	460 511

The number of fungal OTUs detected varied among pecan clonal rootstock genotypes. In the NMU03 group, 365 OTUs were detected in the combined field rhizosphere soil and root samples, while 344 OTUs were identified in the corresponding greenhouse samples. NMU04 showed a markedly higher abundance, with 34,676 OTUs in the field root and rhizosphere soil samples and 44,832 OTUs in the corresponding greenhouse root and rhizosphere soil samples. Similarly, in NMU05, 39,618 OTUs were detected in field samples and 56,481 OTUs in greenhouse samples. For NMU155, field samples harbored 31,430 OTUs, while 24,953 OTUs were identified in the corresponding greenhouse samples.

The aggregated family of bacteria in the NMU03 group yielded a total of 271 OTUs in the root and rhizosphere soil samples from the field, whereas 143 OTUs were identified in the corresponding greenhouse samples (**Table 2**). For the NMU04 group, 695 OTUs were observed in field samples compared to 564 OTUs in greenhouse samples. In the NMU05 group, field-derived samples exhibited 714 OTUs, while greenhouse samples harbored 547 OTUs. Lastly, for the NMU155 group, 695 OTUs were detected in the field samples, compared to 525 OTUs in the greenhouse samples. Aggregated family-level fungal taxonomy within the NMU03 group yielded 170 OTUs in root and rhizosphere soil samples collected from the field, while 130 OTUs were identified in the corresponding greenhouse samples. In the NMU04 group, 385 OTUs were observed in field samples compared to 454 OTUs in greenhouse samples. Similarly, the NMU05 group contained 469 OTUs in field-derived samples and 567 OTUs in greenhouse-derived samples. For the NMU155 group, 460 OTUs were detected in field samples, whereas 511 OTUs were detected in greenhouse samples.

### Rhizosphere soil and roots of pecan clonal rootstocks reveal selective microbial recruitments

The microbial communities associated with greenhouse and field samples of various pecan clonal rootstock genotypes were characterized using high-throughput bacterial and fungal amplicon sequencing. Subsequent taxonomic profiling of the different pecan genotypes revealed distinct yet overlapping family-level microbiomes shared across the clonal rootstock genotypes. In NMU03, among the bacterial families detected, *Burkholderiaceae* was the most abundant in greenhouse roots, accounting for 25% of the total abundance, followed by *Rhodanobacteraceae* (**Supplementary Figure 1A**). Other dominant families in the greenhouse roots included *Chitinophagaceae, Nocardioidaceae*, and *Thermomonosporaceae*, which were also present in the potting mix. However, members of the *Burkholderiaceae* and *Rhodanobacteraceae* families were absent from the field samples, where the *Xanthobacteraceae* dominated, accounting for approximately 10% of the total abundance, followed by the *Nocardioidaceae*. In the NMU04, members of unknown bacterial families formed the most dominant bacterial community in the greenhouse samples, while the field samples exhibited a more diverse bacterial composition with a higher abundance of unknown families and *Haliangiaceae* in the field roots, with a unique presence of *Pedosphaeraceae* in the field soil (**Supplementary Figure 1B**). For NMU05, *Geminicoccaceae* and *Polyangiaceae* were detected across greenhouse and field samples, with the highest abundance of *Geminicoccaceae* in field soil. While NMU05 greenhouse samples were rich in *Rhodothermaceae, Pedosphaeraceae* was detected in field samples. In contrast to greenhouse samples, field samples were dominated by *Rhodothermaceae*, which were absent in greenhouse samples (**Supplementary Figure 1C**). In NMU155, the greenhouse root samples were largely shaped by uncultured bacteria taxa, followed by *Rhodothermaceae* and *Solimonadaceae*, while *Intrasporangiaceae* and an unknown family dominated the field roots sample. *Haliangiaceae* was present in all the sample types; however, *Solimonadaceae* was unique to greenhouse roots, while *Burkholderiaceae* was unique to field roots, and Geminicoccaceae was only detected in the field soil (**Supplementary Figure 1D**).

At the family level, the fungal community revealed a consistent core presence of *Aspergillaceae* across all clonal rootstock genotypes (**Supplementary Figures 2A–D**). In NMNU03, *Aspergillaceae*, *Myxotrichaceae, Pseudeurotiaceae*, and members of unidentified fungal taxa were most abundant in greenhouse roots and the potting mix. In the field, *Pyronemataceae* accounted for approximately 20% of the total soil abundance compared to field roots, whereas *Myxotrichaceae* was more abundant in the field roots than in the soil. In NMU04, greenhouse samples were predominantly characterized by *Aspergillaceae, Myxotrichaceae*, and unidentified fungal taxa, with *Pyronemataceae* being abundant in the potting mix. Despite the higher presence of unidentified fungal communities in NMU04 field samples, *Herpotrichiellaceae* accounted for approximately 11% of the field root abundance. In NMU05, greenhouse root samples showed a richer abundance of *Myxotrichaceae*, *Leotiaceae*, and *Hypocreaceae*, while in the field, *Myxotrichaceae* accounted for approximately 65% abundance of the root-associated mycobiome, followed by *Pyronemataceae* (~23%) and *Hypocreaceae*. Field soil contains the same families of fungi as the roots, suggesting recruitment of these taxa from the soil to the roots. However, both *Elaphomycetaceae* and *Trichocomaceae* were detected in greenhouse samples but were absent in field samples. In NMU155, root-associated fungal communities were largely dominated by unidentified taxa, followed by *Myxotrichaceae* and *Aspergillaceae*. Field roots and field soil exhibited broadly comparable profiles; However, field roots were mainly dominated by *Chaetomiaceae* and *Aspergillaceae*, whereas field soil was *rich* in *Pyronemataceae*, which was not detected in the other samples.

**Figure 2:**
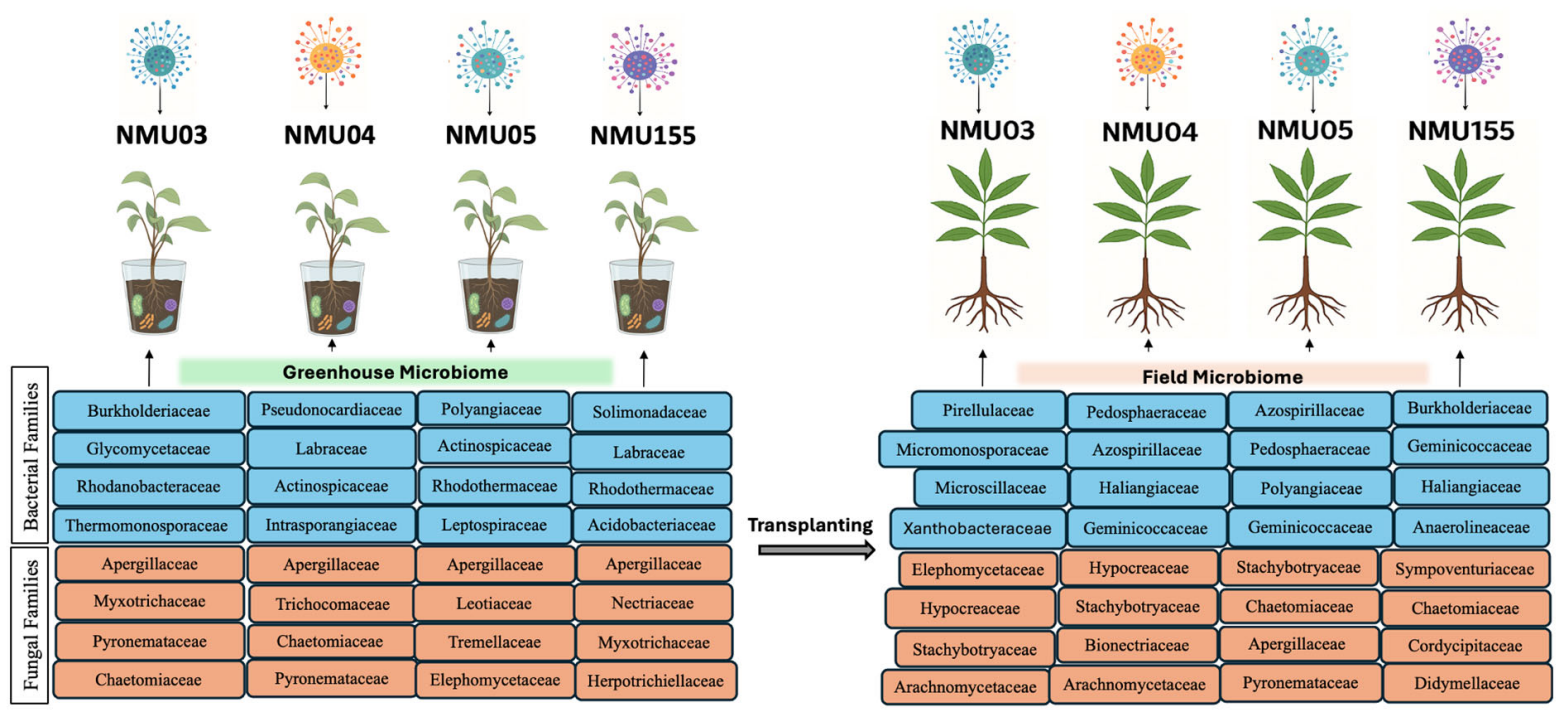
Comparative abundance of bacterial (16S rRNA) and fungal (ITS2) microbiomes based on abundance. Microbiomes associated with the roots of four pecan (*Carya illinoinensis*) clonal rootstocks (NMU03, NMU04, NMU05, and NMU155) grown under greenhouse and field conditions. The colored stacked panels highlight the most abundant microbial families detected in root-associated communities, showing differences between bacterial and fungal assemblages across environments. Tree image credit: https://chatgpt.com/ and https://www.biorender.com/.

Root-associated microbiomes more closely reflect plant-driven selection and host-microbe interactions, while the rhizosphere soil communities provide a broader view of environmental microbial diversity across the investigated clonal rootstock genotypes. A taxonomic profile of the root-associated microbiome unique to pecan revealed genotype-specific microbial assemblages recruited by each clonal rootstock in both greenhouse and field environments (**Figure 2**).

### Alpha diversity by environment and sample types

**Figure 3:**
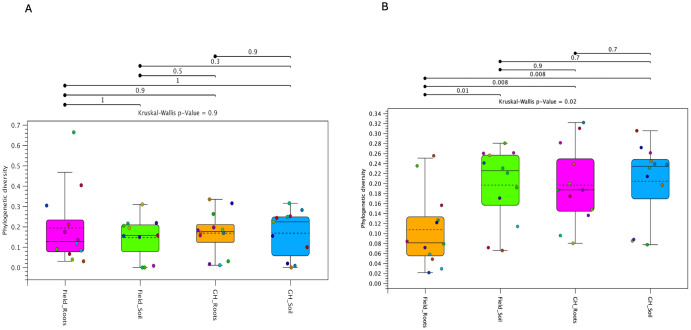
Phylogenetic alpha diversity of pecan clonal rootstocks (NMU03, NMU04, NMU05, and NMU155) from the greenhouse and the field. The box and whisker plots show **(A)** Bacterial and **(B)** Fungal communities across pecan roots and rhizosphere soil samples from field and greenhouse environments. Colored dots represent seedling samples per tissue type. The individual dotted samples in each sample group indicate a relationship of homogeneity across the groups. Keys: Field_Roots (Field Roots), Field_Soil (Field Soil), GH_Roots (Greenhouse roots) and GH_Soil (Greenhouse Soil).

Phylogenetic alpha diversity of bacterial communities across field and greenhouse samples for the four genotypes showed no statistically significant differences (Kruskal-Wallis p = 0.9) among the four sample groups (field roots, field soil, greenhouse roots, and greenhouse soil) (**Figure 3A**). However, the alpha diversity of the fungal microbiome varied significantly among the four sample groups, as indicated by a significant Kruskal–Wallis test (p = 0.02). Pairwise comparisons revealed a significant difference between greenhouse and field-grown roots (p = 0.008). Furthermore, field roots exhibited significantly lower diversity than field soil, greenhouse roots, and greenhouse soil (*p* < 0.01) (**Figure 3B**).

### Beta diversity by environment and sample types

**Figure 4:**
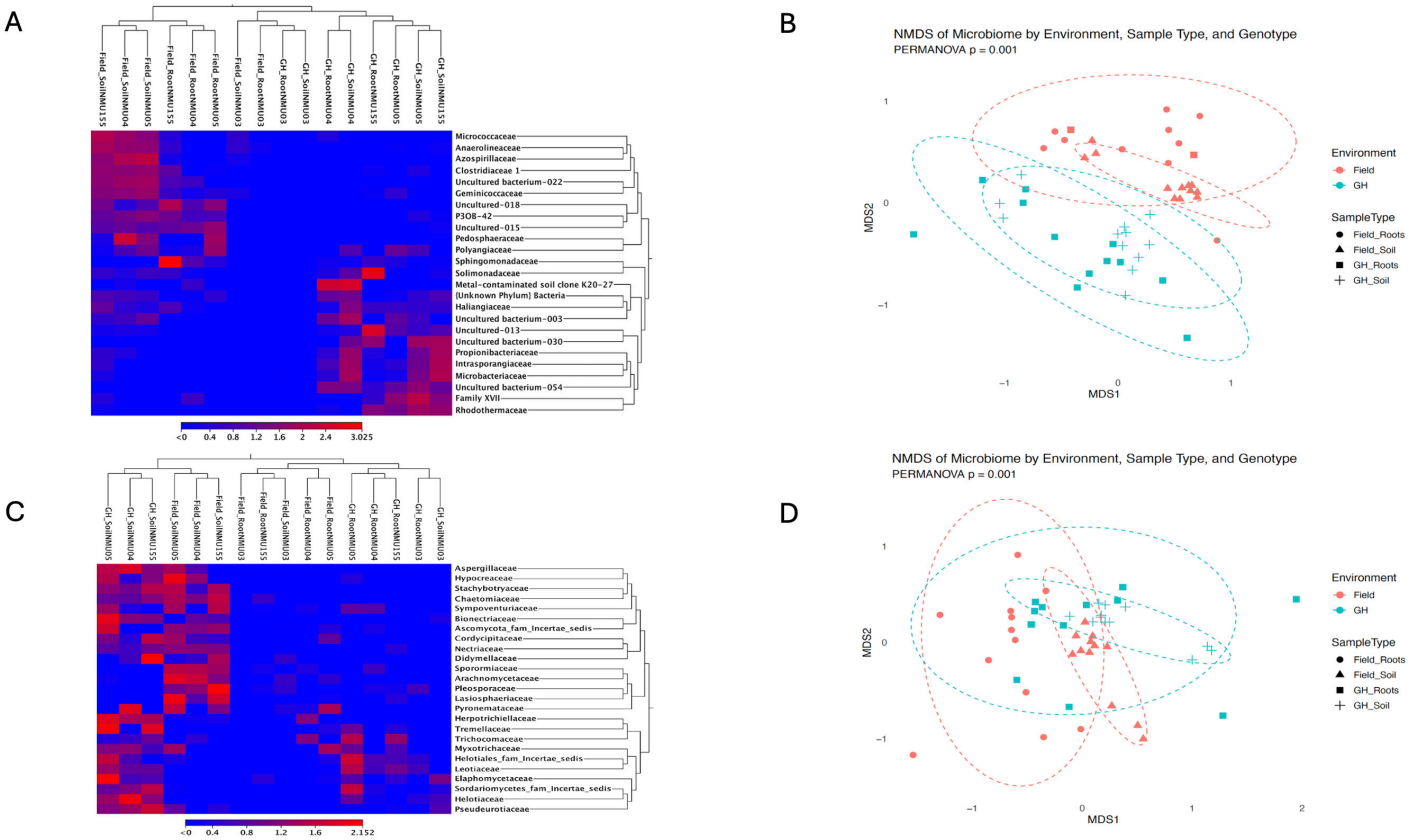
Bacterial and fungal populations in pecan clonal rootstocks (NMU03, NMU04, NMU05, and NMU155) from the greenhouse and the field. **(A)** Heatmap of Bacterial family-level composition across root and soil samples from greenhouse (GH) and field environments **(B)**. NMDS visualization of bacterial communities grouped by environment, sample type, and genotype with stress metric = 0.184 **(C)**. Heatmap of Fungal family-level composition across the same compartments and environments **(D)**. NMDS visualization of fungal communities, highlighting distinct clustering by sample type and genotype with stress metric = 0.171. In both heatmaps, columns represent individual samples categorized by environment (GH or Field), sample group (Soil or Root), and genotypes (NMU03, NMU04, NMU05, NMU155). In contrast, rows represent fungal families clustered by relative abundance. The color gradient (scale: <0 to 3.025). Eclipse depicts 95% confidence intervals around group centroids, illustrating the multivariate dispersion of samples within each environment. Limited overlap between eclipses indicates distinct microbial community structures between greenhouse and field samples, as confirmed by PERMANOVA (p= 0.001).

Bacterial community structure across field and greenhouse samples revealed distinct taxonomic and compositional differences, driven by both environmental factors and genotype. The bacterial abundance heatmap showed that several families were differentially enriched across sample types (**Figure 4A**). Clustering patterns in the heatmap further highlighted the grouping of samples by environment and compartment, reflecting shared microbial profiles within these categories. These taxonomic trends were corroborated by non-metric multidimensional scaling (NMDS) of beta diversity (**Figure 4B**), which suggested differentiation between field and greenhouse bacterial communities, although some overlap between groups was observed (**Supplementary Figure 3**). PERMANOVA testing confirmed that these differences were statistically significant (p = 0.001), indicating strong environmental structuring of microbial communities. Further analysis revealed that the ordination of field-root bacterial samples tended to occur more often on the positive side of NMDS2, whereas greenhouse samples were more often located on the negative side, although overlap between groups was observed (**Supplementary Figure 3A**). Within each environment, samples exhibited partial clustering by genotype; however, these results suggest that both environmental conditions and genotype primarily structure root microbiomes. PERMANOVA of root bacteriome confirmed that environment (*R^2^ = 0.*141*, P = 0.001*) and host genotype (*R^2^ = 0.*203*, P = 0.001*) significantly influenced soil microbial community composition (**Supplementary Table 1**). However, the environment-genotype interaction was not significant within the root bacterial community (*R^2^ = 0.124, P = 0.125*), and the residual effects accounted for 53% of the unexplained community variation (R2 = 0.533).

Soil fungal communities exhibited distinct clustering patterns based on environment (field vs. greenhouse) and sample type (root vs. soil) as revealed by both taxonomic and NMDS analyses. A heatmap of fungal family abundance likewise revealed the compartmental enrichment of several fungal families across environments and sample types (**Figure 4C**). This compartmental enrichment was supported by NMDS analysis, which suggested differentiation of fungal communities by environment and, to a lesser extent, sample type (**Figure 4D**). Field and greenhouse samples occupied different regions of the ordination space, with substantial overlap between groups. Similarly, root- and soil-associated communities were not clearly separated in the ordination. Despite this overlap, PERMANOVA indicated that community composition differed significantly among groups (p = 0.001). Further analysis revealed a partial separation of root fungal communities between field and greenhouse environments (**Supplementary Figure 3B**). This indicates that environmental conditions contribute to the structuring of the fungal microbiome. However, substantial overlap among genotypes and environments suggests that fungal communities were more heterogeneous and less strongly structured than bacterial communities. PERMANOVA of root fungal communities revealed that both environment (*R² = 0.089, P = 0.011*) and genotype (*R² = 0.209, P = 0.003)* influenced microbial community composition, with a significant interaction effect (*R² = 0.173, P = 0.013*). These results also suggested that the residual effects accounted for 53% (*R^2^ = 0.5*29) of the unexplained community variation (**Supplementary Table 2**).

### Alpha diversity by genotype and sample type

Phylogenetic alpha diversity of bacterial community among the different genotypes (NMU03, NMU04, NMU05, and NMU155) revealed differences in microbiome diversity (**Supplementary Figures 4A–D**). Alpha diversity of the bacterial community, compared across the NMU03 samples from different environments, revealed significant phylogenetic diversity among the groups (Kruskal-Wallis, P = 0.03). Field roots had the most diverse bacterial community in the NMU03 group, followed by greenhouse roots, while the least diverse groups were the field and greenhouse soil. In contrast, the alpha diversity of the fungal community, compared across the NMU03 sample groups from different environments, revealed no pairwise phylogenetic diversity among the groups (Kruskal-Wallis p-value=0.3), indicating a relatively stable fungal microbiome composition across environments (field and greenhouse) for the NMU03 sample groups (roots and soil).

NMU04 bacterial and fungal alpha diversity across sample groups from different environments showed no statistically significant overall difference (Kruskal-Wallis p-value for both: *p* = 0.2). Furthermore, phylogenetic alpha diversity of bacterial population was compared across the NMU05 groups under both greenhouse and field conditions, and no statistically significant overall difference was observed (*p* = 0.7). This indicates a relatively stable bacterial microbiome composition across both environments in the NMU05 genotype. The phylogenetic alpha diversity of the fungal community, compared across the NMU05 groups from these environments, also indicated no statistically significant overall difference (*p* = 0.1) (**Supplementary Figures 5A–D**). Likewise, the alpha diversity of NMU155 showed no statistically significant difference in the bacterial community across groups in either environment (Kruskal-Wallis p-value = 0.8), and the phylogenetic alpha diversity for the fungal population among the NMU155 groups was also not statistically significant (Kruskal-Wallis p-value = 0.8), and the phylogenetic alpha diversity of the fungal population among the NMU155 groups was also not statistically significant (p-value = 0.08). Alpha diversity did not differ significantly among groups for NMU155 (Kruskal–Wallis, p = 0.8), whereas cumulative OTU richness varied by environment and compartment. Bacterial and fungal OTU counts were lowest in field roots and higher in greenhouse roots, while total soil OTUs were comparable between field and greenhouse samples.

## Discussion

### Environments and rootstock genotypes shaped microbiome composition

Our study emphasizes the importance of environmental conditions and host genotype in shaping the composition and diversity of bacterial and fungal communities associated with the roots and rhizosphere soils of pecan clonal rootstocks, both in greenhouse conditions and in their corresponding subsets transplanted to the field for over 2 years. These findings provide insight into early-stage microbiome assembly and plant-microbe interactions that may influence long-term tree performance, rather than conclusive predictors of microbiomes in established orchards. Previous studies of Pomelo trees across multiple developmental stages, through high-throughput Illumina MiSeq sequencing, have demonstrated that soil microbial communities undergo pronounced and dynamic shifts with increasing tree age (Zheng et al., 2022). Likewise, studies on grapevine-associated microbiota have revealed the influence of clonal rootstock genotypes on the recruitment and assemblage of rhizosphere and root endosphere microbiomes (ZarraonaIndia et al., 2015; Darriaut et al., 2022; Vink et al., 2021). Notably, as measured by the number of OTUs in rhizosphere soil and greenhouse-collected roots of these clonal rootstocks, bacterial and fungal communities were consistently higher in field-derived samples than in those collected from greenhouse samples. This pattern was observed across all four studied pecan rootstock genotypes, suggesting that the field environment has a significant impact on microbiome assemblage. This finding is consistent with previous observations that field environments, with more complex ecological dynamics and heterogeneous climatic factors, support more diverse microbial populations than the relatively controlled and homogeneous conditions of greenhouse environments (Fierer and Jackson, 2006; Williams and Marco, 2014).

OTUs were markedly higher for fungi than for bacteria across all genotypes. The possibility that pecans lack root hairs and form a mutualistic association with mycorrhizal fungi, which serves as a functional alternative to root hairs to enhance nutrient uptake, cannot be disregarded (Woodroof and Woodroof, 1934). Roots colonized by mycorrhizal fungi may harbor dense and diverse fungal communities, leading to elevated fungal OTU counts. Based on our OTU tables, *Elaphomycetacae* and *Pyronemataceae* make up nearly 35% of the abundance in some clonal rootstock samples. In addition, in some samples we see nearly 75% of the abundance attributed to ‘unidentified’ families (data not provided). As we have visually observed sampled roots clearly associated with mycorrhizal fungi (data not shown) we assume that some of these taxa were these identified families. However, we have not cultured to confirm this suspicion. We also recognize that our DNA extraction method may not adequately capture all fungal organisms associated with root and soil microbiomes (Stock et al., 2025). The *Elaphomycetaceae* family is known to establish ectomycorrhizal relationships with diverse Angiosperms and Gymnosperms (Fuente et al., 2023; Castellano and Stephens., 2017). Additionally, since the samples were collected during the fall season, relatively cooler temperatures and reduced root activity at this time of year could have contributed to the lower bacterial abundance observed, as bacterial populations are often more sensitive to temperature and seasonal changes than fungi (Pietikäinen et al., 2005; Wan et al., 2024; Duque-Zapata et al., 2025). A similar trend of fungal dominance was reported from a greenhouse study where seedlings from four different maternal pecan cultivars (*Mahan, ‘Pawnee’, ‘Wichita’*, and *‘Western’*) exhibited higher fungal OTUs (428,370) compared to the 5,603 bacterial OTUs detected (Cervantes et al., 2022).

Distinct microbial assemblages were also detected between the rhizosphere soil and root endosphere within each genotype, suggesting niche specialization and selective recruitment by the host. Interestingly, NMU03 exhibited lower microbial abundance at the OTU level but supported broader taxonomic diversity at the family level, indicating selective recruitment of diverse functional groups. This pattern may reflect genotype-specific shaping of the microbiome. Previous studies on pecan have identified a core microbiome as a group of microbial taxa consistently associated with a particular host or environment (Cervantes et al., 2022; Ren et al., 2024). This study’s detection of shared microbial taxa across clonal rootstock genotypes suggests that pecan rootstocks maintain a characteristic microbiome signature that persists across greenhouse and field environments. These shared taxa may play key functional roles, such as nutrient acquisition and disease suppression; however, further investigations are needed to clarify their ecological significance.

### Selective recruitment of bacteriomes by pecan clonal rootstocks reveals genotype-specific microbiomes

In the NMU03 group, greenhouse root samples displayed lower overall bacterial abundance and were dominated by a few families, particularly *Burkholderiaceae* and *Rhodanobacteraceae.* In contrast, field root samples exhibited a more diverse bacterial community, with the *Xanthobacteraceae* family emerging as the dominant group. This dominance may reflect the ecological role of specific genera within this family, such as *Azorhizobium*, which are known for their ability to fix atmospheric nitrogen and contribute to soil nitrogen enrichment (Yang et al., 2020). The presence of *Burkholderiaceae, Microscillaceae*, and *Glycomycetaceae* aligns with a previous report identifying these taxa as signature members of the core microbiome in greenhouse-propagated pecan seedlings (Cervantes et al., 2022). Although most of the signature core microbiome previously reported by Cervantes et al. (2022) in greenhouse-propagated pecan seedlings was not dominant in this study, this observation likely reflects shifts in relative abundance rather than a complete loss. Such changes may result from environmental transitions as pecans are transferred from greenhouse to field conditions, suggesting a later successional fate of these core microbiome members. Similarly, *Pseudonocardiaceae*, previously identified in the diverse microbiomes of pecan genotypes from a tissue culture study (Randall et al., 2021), was also detected in the greenhouse roots of NMU04. Members of this family are well known for producing bioactive secondary metabolites, including antibiotics, and for contributing to plant defense against pathogens through symbiotic interactions (Golinska et al., 2015). Their persistence in tissue culture and pecan-associated root microbiomes indicates an ecologically significant role in enhancing host resilience and maintaining root health under greenhouse conditions.

The consistent detection of *Burkholderiaceae* and *Xanthobacteraceae* across both environments further highlights their potential functional importance, as these families have also been reported in the inner root regions of cultivated organic tea, where they are linked to enhanced metabolic activity (Chen et al., 2021; Bag et al., 2022). In the NMU04 group, greenhouse roots and soil were strongly dominated by a single unclassified bacterial family, which accounted for approximately 30% of the relative abundance, suggesting ecological dominance under controlled conditions. In contrast, *Geminicoccaceae* and *Pedosphaeraceae* dominated field soil and were detected within the diverse bacteriome of field root compartments; however, they were less represented in the greenhouse bacterial community. The unique presence of *Pedosphaeraceae* in field soil may be associated with its importance in soil restoration and its ability to stabilize ecosystems following disturbance (Zhang et al., 2019; Li et al., 2021). A similar pattern was observed in NMU05, where *Pedosphaeraceae* was dominant in the field roots. This further supports its ecological role in soil restoration within natural environments.

Similarly, in NMU155, *Haliangiaceae* emerged as one of the dominant families in field samples but was less represented in greenhouse samples, suggesting an adaptation to the prevailing field environment. However, greenhouse root samples were dominated by a single uncultured bacterial family, accounting for more than 40% of the relative abundance, a pattern similar to that observed in NMU04 greenhouse samples. However, field root and soil compartments showed a broader taxonomic distribution than the more restricted assemblages observed under greenhouse conditions. The bacterial communities comprised *Geminicoccaceae*, *Rhodothermaceae*, *Intrasporangiaceae*, and *Haliangiaceae*, whose distributions are likely shaped by complex biotic and abiotic interactions within the natural environment (Fierer and Jackson, 2006). *Geminicoccaceae* was dominant across most genotypes, except in the NMU03 clonal rootstock, suggesting potential genotype-specific influences on bacterial recruitment. The high proportions of unclassified and uncultured taxa across NMU04, NMU05, and NMU155 samples, especially in greenhouse samples, highlight the need for improved taxonomic classification to fully understand their ecological roles in the pecan rhizosphere and root microbiomes.

### Selective recruitment of mycobiomes by pecan clonal rootstocks reveals genotype-specific microbiomes

The fungal community profiles across pecan clonal rootstock genotypes revealed strong genotype and environment-specific structuring at the family level. It is important to note that some of the fungal species identified may have been introduced while grown in the greenhouse either from the commercial potting mix or from the greenhouse environment. *Aspergillaceae* emerged as the predominant fungal family in all rootstocks across sample types in addition, this family was previously identified in pecan seed microbiome (Cervantes et al., 2022). This suggests its ecological dominance and potential adaptation to both root and rhizosphere niches under varied environmental conditions. Notably, members of the *Aspergillaceae* family have been reported to enhance plant tolerance to water deficit conditions through the biosynthesis of specific metabolites, including (Z)-N(4-hydroxystyryl) formamide (NFA), as well as various phytohormones (Hung and Lee Rutgers, 2016; Qin et al., 2019; Fuentes et al., 2020). This further suggests that both genotypic and environmental factors may shape the prevalence of this family. This dominance may also reflect a competitive advantage in nutrient acquisition or tolerance to environmental stresses commonly encountered in natural environments (Fierer and Jackson, 2006). In the NMU05 group, dominance by the fungal families *Aspergillaceae, Elaphomycetaceae*, and *Myxotrichaceae* was observed. The presence of *Elaphomycetaceae*, a family known for ectomycorrhizal associations, alongside saprotrophic *Aspergillaceae* and *Myxotrichaceae* could indicate functional complementarity within the fungal microbiome of this genotype (Castellano and Stephens., 2017; Fuente et al., 2023). This co-dominance suggests that NMU05 supports a diverse fungal microbiome, facilitating nutrient mobilization and host colonization. The fungal assemblage in NMU155 was notably distinct, with field root samples supporting a more varied fungal community, including members of the *Aspergillaceae*, *Chaetomiaceae*, and *Didymellaceae* families. These taxa are known for their saprotrophic and endophytic capabilities, suggesting a more functionally resilient fungal community under field conditions (Chen et al., 2017; Leetanasaksakul et al., 2024; Tidimalo et al., 2024). These findings highlight the dynamic and compartment-specific nature of fungal community assembly in pecan rootstocks, influenced by a combination of host genotype, environmental conditions, and niche differentiation. The repeated dominance of *Aspergillaceae* across genotypes and compartments suggests a central ecological role in the pecan rhizosphere. At the same time, unclassified taxa highlight the need for further taxonomic and functional classification within the fungal microbiome.

### Rhizosphere, root compartments, and environment-shaped microbial diversity in pecan clonal rootstock

Alpha diversity of bacterial taxonomy remained relatively stable across genotypes and sample types, whereas fungal composition showed significant variation between field and greenhouse roots. This variation suggests that although fungal abundance was higher than bacterial abundance across all genotypes, the field root-associated fungal microbiome exhibited lower fungal diversity than that observed in greenhouse samples. Such patterns highlight the influence of environmental conditions in shaping the composition and diversity of soil fungal communities (Fierer and Jackson, 2006). Across both bacterial and fungal communities, NMDS ordinations revealed clear separation between field and greenhouse samples as well as distinct clustering between soil and root compartments, indicating that environmental conditions and plant compartments are key drivers of microbiome composition. These patterns were supported by PERMANOVA analyses, which showed that both environment and host genotype significantly influenced microbial community structure in soil and roots, with genotype explaining a larger proportion of variation in soil communities. Furthermore, significant environment × genotype interactions suggest that genotype-associated microbial recruitment may vary depending on environmental conditions. The clear compartmental separation observed in the NMDS plots, particularly between root and soil bacterial communities, is consistent with findings from other perennial cropping systems where plant roots act as selective microbial habitats that filter microbial taxa from the surrounding soil microbiome (ZarraonaIndia et al., 2015; Darriaut et al., 2022; Vink et al., 2021). Together, these results indicate that microbiome assembly in pecan clonal rootstocks is shaped by the combined influence of environment, plant compartment, and host genotype, with environmental conditions exerting a dominant effect.

## Conclusion

Findings from this study highlight the role of host genotype in shaping root-associated microbiomes and their responses to environmental variation.

The observed genotype-specific differences in bacterial and fungal alpha diversity across field and greenhouse environments suggest that genotypes of pecan clonal rootstock genotypes modulate microbiome assembly, particularly within the root compartment. Roots consistently exhibited greater sensitivity to environmental conditions than soil, highlighting their role as selective microbial niches influenced by abiotic and host (genotype) driven factors. These genotype-environment interactions will guide microbiome-informed breeding strategies to optimize plant-microbe associations under variable growth conditions among pecan breeders. By leveraging plant genotype and environmental conditions, further research will be conducted on the mechanism of microbiome recruitment to pecan roots and their functional roles at different growth stages. This will enable the tracking of microbial community succession from establishment through nut-bearing stages to fully understand microbiome development across the pecan life cycle. This could also reveal how the microbiome enhances nutrient acquisition, improves stress tolerance, and promotes plant health in pecan trees. These insights will deepen our understanding of microbiome assembly in perennial crops and support the development of targeted strategies for sustainable agriculture through effective root microbiome management.

## Data Availability

The data presented in this study are deposited in the NCBI SRA with the accession numbers PPRJNA1293544 (bacterial) and PRJNA1294893 (fungal). https://www.ncbi.nlm.nih.gov/bioproject/.

## Supplementary material

Supplementary Table 1 PERMANOVA results evaluating the effects of environment, genotype, and their interaction on bacterial community composition associated with pecan rootstocks. Analyses were performed using Bray-Curtis dissimilarity matrices derived from 16S rRNA gene sequencing data.

Supplementary Table 2 PERMANOVA results evaluating the effects of environment, genotype, and their interaction on fungal community composition associated with pecan rootstocks based on ITS sequencing data.

Supplementary Figure 1 Relative abundance of bacterial families detected in the roots and rhizosphere of Pecan clonal rootstock genotypes (NMU03, NMU04, NMU05, and NMU05) under greenhouse and field conditions. Stacked bar plots illustrate bacterial community composition in NMU03 **(A)**, NMU04 **(B)**, NMU05 **(C)**, and NMU155 **(D)**. The relative abundance (%) of each bacteriome is shown on the y-axis Keys: Field_Roots (Field Roots), Field_Soil (Field Soil), GH_Roots (Greenhouse roots) and GH_Soil (Greenhouse Soil).

Supplementary Figure 2 Relative abundance of fungal families detected in the roots and rhizosphere of Pecan clonal rootstock genotypes (NMU03, NMU04, NMU05, and NMU05) under greenhouse and field conditions. Stacked bar plots illustrate bacterial community composition in NMU03 **(A)**, NMU04 **(B)**, NMU05 **(C)**, and NMU155 **(D)**. The relative abundance (%) of each bacteriome is shown on the y-axis Keys: Field_Roots (Field Roots), Field_Soil (Field Soil), GH_Roots (Greenhouse roots) and GH_Soil (Greenhouse Soil).

Supplementary Figure 3 Non-metric multidimensional scaling (NMDS) ordination of root-associated microbial communities based on Bray-Curtis dissimilarity. **(A)** Bacterial communities with stress metric = 0.11 and **(B)** fungal communities associated with pecan rootstocks with stress metric = 0.093. Each point represents an individual sample, colored and shaped according to genotype and environment (field or greenhouse). The ordination illustrates differences in microbial community composition among genotypes and environments. Separation of samples along the NMDS axes indicates variation in community structure, The NMDS stress value indicates the goodness-of-fit of the ordination, representing how well the two-dimensional plot reflects the original multivariate distance relationships among samples.

Supplementary Figure 4 Phylogenetic alpha diversity of bacterial communities in the roots and rhizosphere of pecan clonal rootstocks under greenhouse and field conditions. Box plot illustrates the bacterial diversities in NMU03 **(A)**, NMU04 **(B)**, NMU05 **(C)**, and NMU155 **(D).**

Supplementary Figure 5 Phylogenetic alpha diversity of mycobiomes in the roots and rhizosphere of pecan clonal rootstocks under greenhouse and field conditions. Box plot illustrates the bacterial diversities in NMU03 **(A)**, NMU04 **(B)**, NMU05 **(C)**, and NMU155 **(D).**
