# Supplementary figures and images for: Host genotype and environment shape rhizosphere and root microbiome composition of pecan rootstocks
Source: Front Microbiomes. 2026 May 15;5:1778537. doi: 10.3389/frmbi.2026.1778537 (PMC13219382; doi:10.3389/frmbi.2026.1778537)

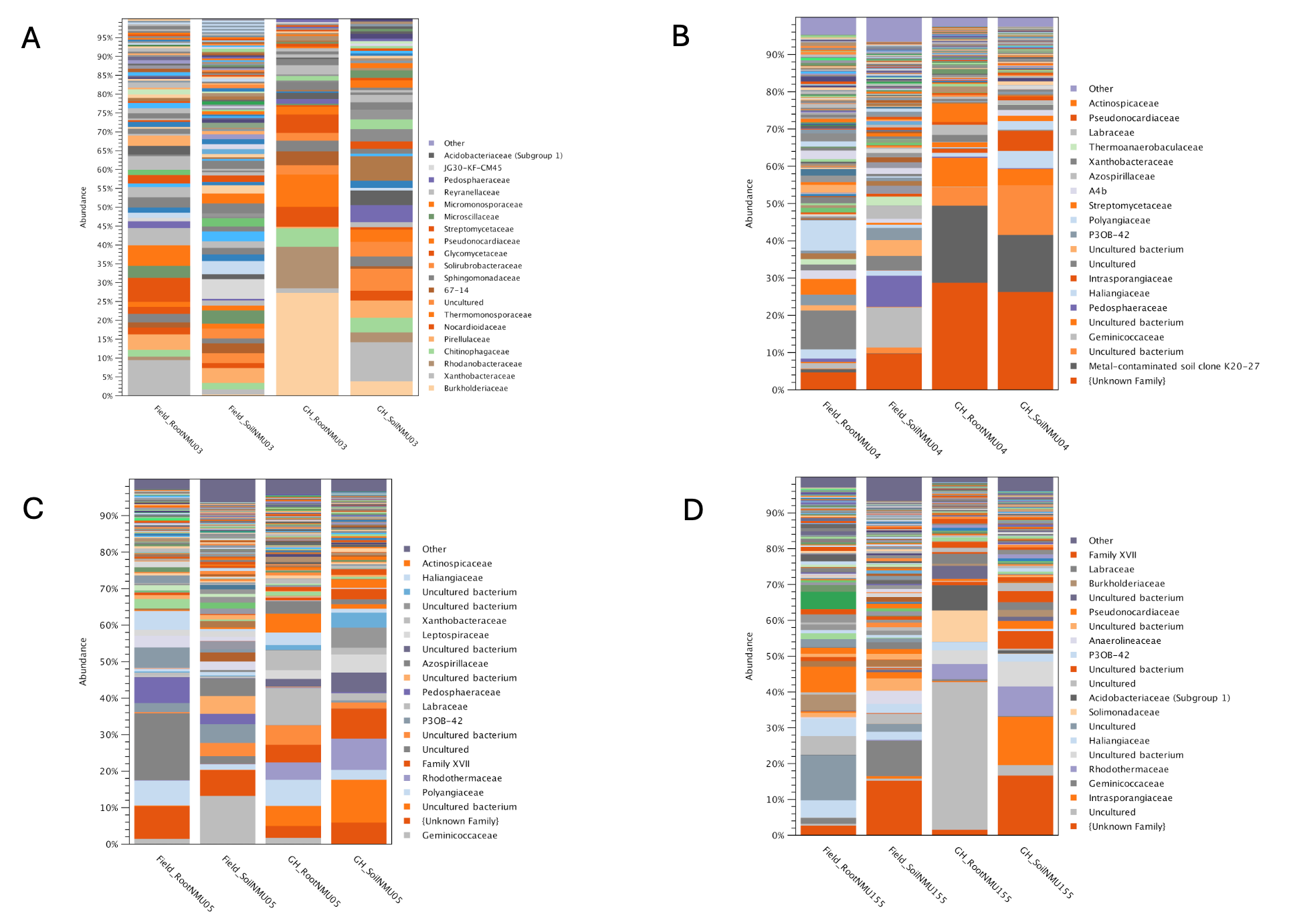

Supplement: Supplementary Figure 1 — Relative abundance of bacterial families detected in the roots and rhizosphere of Pecan clonal rootstock genotypes (NMU03, NMU04, NMU05, and NMU05) under greenhouse and field conditions. Stacked bar plots illustrate bacterial community composition in NMU03 (A), NMU04 (B), NMU05 (C), and NMU155 (D). The relative abundance (%) of each bacteriome is shown on the y-axis Keys: Field_Roots (Field Roots), Field_Soil (Field Soil), GH_Roots (Greenhouse roots) and GH_Soil (Greenhouse Soil). [file Image1.jpeg]

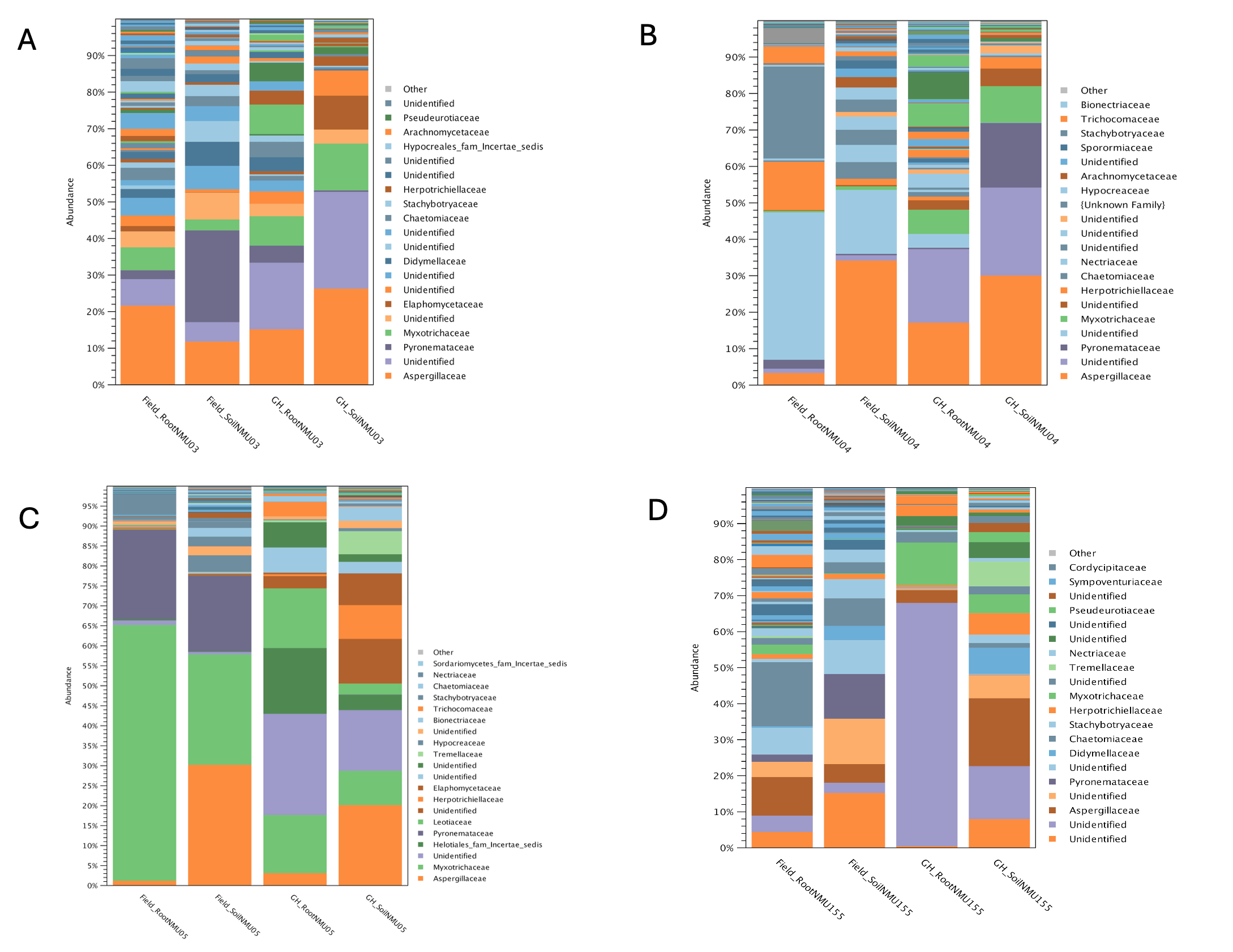

Supplement: Supplementary Figure 2 — Relative abundance of fungal families detected in the roots and rhizosphere of Pecan clonal rootstock genotypes (NMU03, NMU04, NMU05, and NMU05) under greenhouse and field conditions. Stacked bar plots illustrate bacterial community composition in NMU03 (A), NMU04 (B), NMU05 (C), and NMU155 (D). The relative abundance (%) of each bacteriome is shown on the y-axis Keys: Field_Roots (Field Roots), Field_Soil (Field Soil), GH_Roots (Greenhouse roots) and GH_Soil (Greenhouse Soil). [file Image2.jpeg]

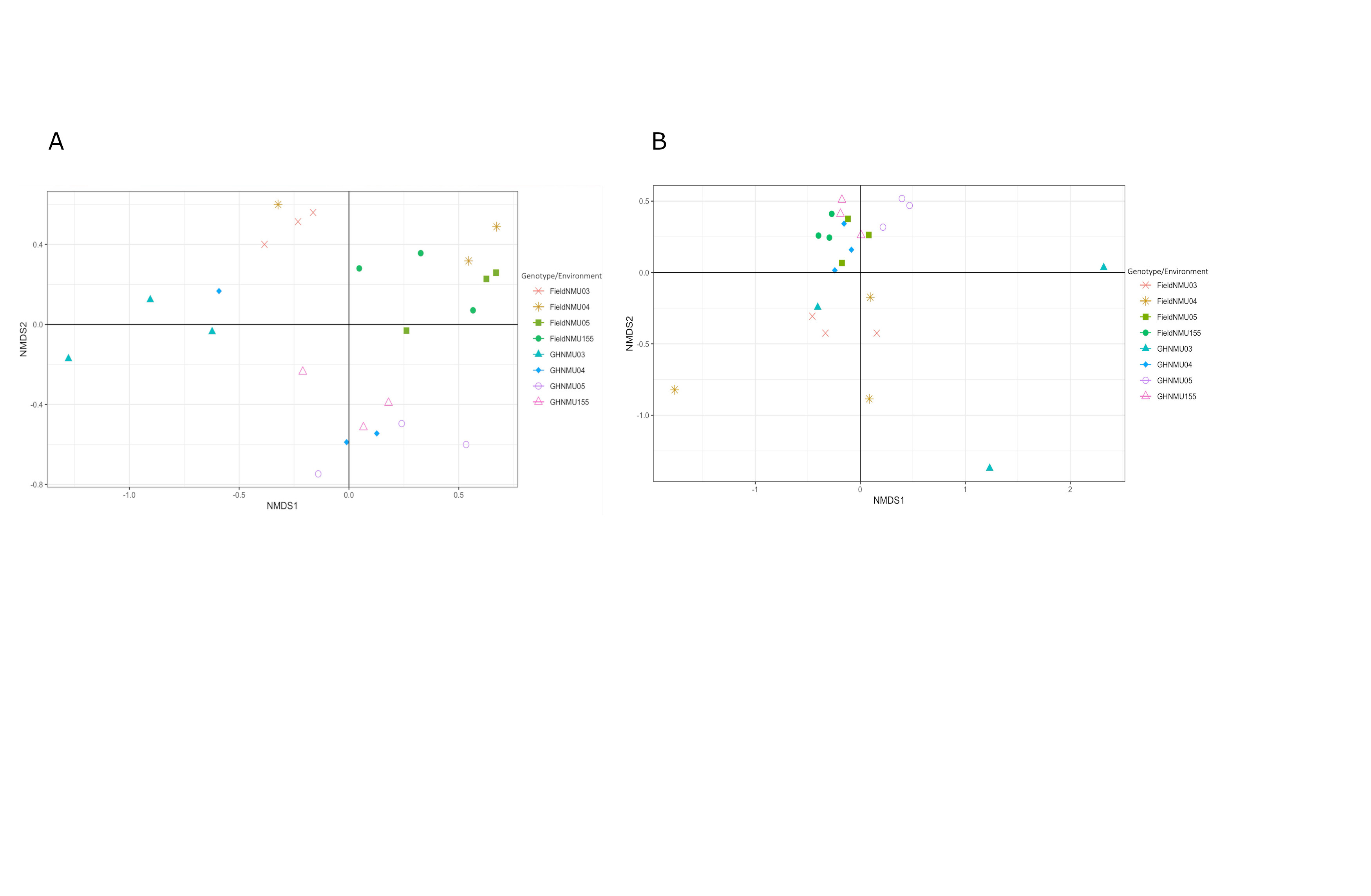

Supplement: Supplementary Figure 3 — Non-metric multidimensional scaling (NMDS) ordination of root-associated microbial communities based on Bray-Curtis dissimilarity. (A) Bacterial communities with stress metric = 0.11 and (B) fungal communities associated with pecan rootstocks with stress metric = 0.093. Each point represents an individual sample, colored and shaped according to genotype and environment (field or greenhouse). The ordination illustrates differences in microbial community composition among genotypes and environments. Separation of samples along the NMDS axes indicates variation in community structure, The NMDS stress value indicates the goodness-of-fit of the ordination, representing how well the two-dimensional plot reflects the original multivariate distance relationships among samples. [file Image3.jpeg]

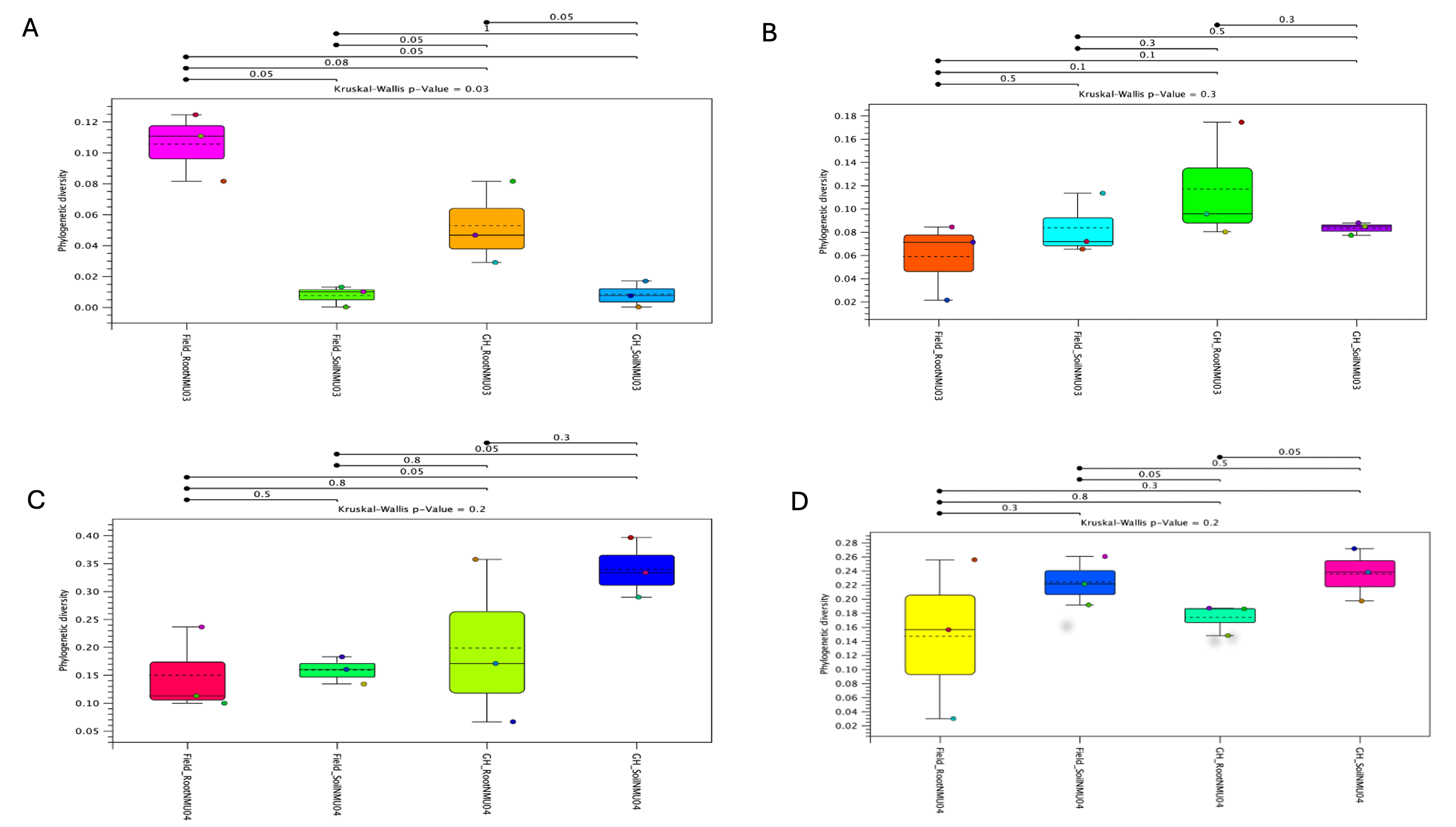

Supplement: Supplementary Figure 4 — Phylogenetic alpha diversity of bacterial communities in the roots and rhizosphere of pecan clonal rootstocks under greenhouse and field conditions. Box plot illustrates the bacterial diversities in NMU03 (A), NMU04 (B), NMU05 (C), and NMU155 (D). [file Image4.jpeg]

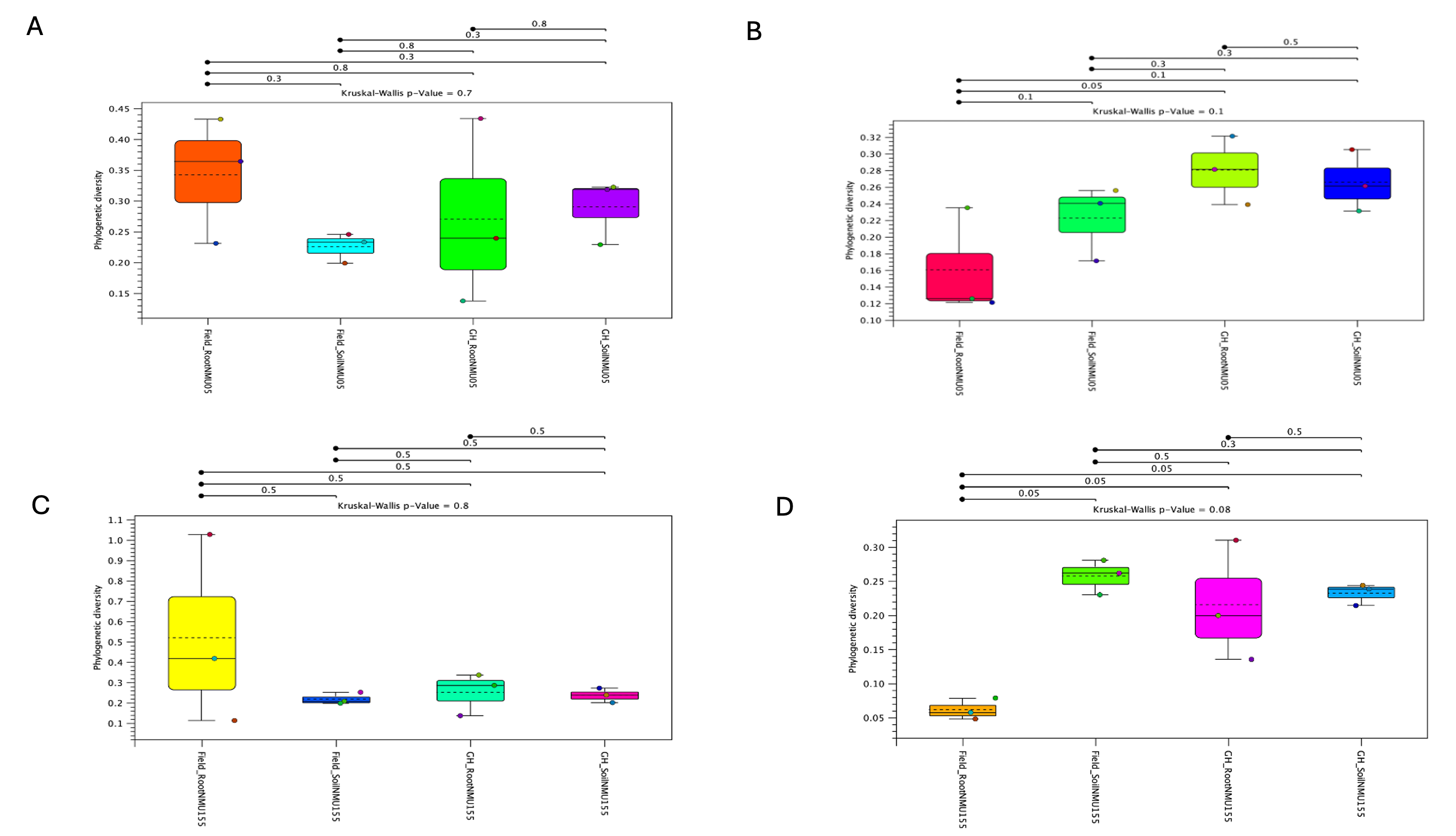

Supplement: Supplementary Figure 5 — Phylogenetic alpha diversity of mycobiomes in the roots and rhizosphere of pecan clonal rootstocks under greenhouse and field conditions. Box plot illustrates the bacterial diversities in NMU03 (A), NMU04 (B), NMU05 (C), and NMU155 (D). [file Image5.jpeg]
